# Microbial Responses to Micronutrient Amendments in Oxygenated and Deoxygenated Waters of the Arabian Sea

**DOI:** 10.1111/1758-2229.70072

**Published:** 2025-05-02

**Authors:** Mandar Bandekar, Rakhee Khandeparker, Kuldeep D. More, Seyieleno C. Seleyi, Mukund Gauthankar, Ujwala Amberkar, Jukka Kekäläinen, Jarkko Akkanen

**Affiliations:** ^1^ Department of Environmental and Biological Sciences University of Eastern Finland Joensuu Finland; ^2^ Biological Oceanography Division CSIR‐National Institute of Oceanography Dona Paula India; ^3^ Department of Biological Sciences State University of New York at Buffalo Amherst New York USA; ^4^ Marine Biotechnology Division National Institute of Ocean Technology, Ministry of Earth Sciences Chennai India; ^5^ Départment des Sciences Fondamentales Université du Québec à Chicoutimi Chicoutimi Québec Canada; ^6^ Environmental Impact Assessment Division National Centre for Polar and Ocean Research Vasco‐da‐Gama India

**Keywords:** Arabian Sea, microbial communities, microcosm experiment, micronutrients, oxygen minimum zone

## Abstract

Metalloenzyme cofactors and oxygen conditions are crucial for microbial metabolism, yet their combined effects on microbial ecosystems remain unexplored. This study explores the impact of micronutrient amendments (Zn, Fe, Co and their combinations) on the microbial community composition in oxygenated (73 m) and deoxygenated (200 m) waters of the Arabian Sea. Through controlled microcosm experiment and 16S rRNA amplicon sequencing, we observed that micronutrients significantly alter nutrient concentrations and microbial dynamics. At 73 m, micronutrient treatments reduced nitrate, nitrite and ammonia levels, whereas at 200 m, they increased nitrate and silicate levels. Total bacterial counts (TBCs) were higher in all treatments at both depths, with Fe showing the highest counts. Alpha diversity indicated that Fe‐amended flask increased microbial diversity the most at 73 m, while mixed treatments had a pronounced effect at 200 m. Taxonomic analysis revealed significant genus‐level variations in both bacteria and archaea. One‐way analysis of variance (ANOVA) confirmed micronutrient impacts on nutrients and TBC. Canonical correspondence analysis (CCA) and non‐metric multidimensional scaling (NMDS) revealed distinct clustering based on oxygen conditions. These results confirm our hypothesis that micronutrient amendments in varying oxygen levels distinctly alter microbial community composition and nutrient cycling in marine environments.

## Graphical abstract

Microcosm experiments and 16S rRNA amplicon sequencing show that micronutrients (Zn, Fe, Co) or their combination (Zn + Fe + Co) significantly influence nutrient concentrations and microbial dynamics in both oxygenated (73 m) and deoxygenated (200 m) waters of the Arabian Sea. Our findings highlight that Fe is particularly impactful, enhancing microbial diversity and total bacterial counts, especially in oxygenated conditions, while mixed micronutrient treatments had the most significant effects in deoxygenated depth.

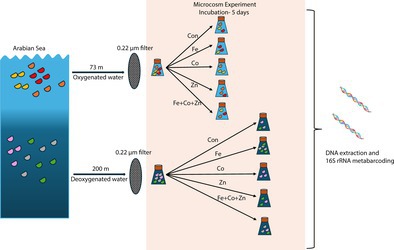

## 1 Introduction

Microorganisms are crucial in marine environments contributing significantly to ecological processes and global biogeochemical cycles. They encompass diverse taxa that collectively shape the marine ecosystem dynamics (Falkowski, Fenchel, and Delong 2008). Marine microorganisms are crucial for nutrient cycling, as they participate in processes such as nitrogen fixation, denitrification and carbon sequestration (Azam and Malfatti 2007). Autotrophic microbiota is also crucial for primary production through photosynthesis contributing significantly to global oxygen (O_2_) production (Worden et al. 2015). Furthermore, marine microbiota plays a vital role in enabling ecosystem functions by forming symbiotic relationships with other aquatic organisms, both macro and micro, across different trophic levels, thereby sustaining nutrient exchange (Wommack and Ravel 2013).

The structure and composition of microbial communities are influenced by various environmental factors, including micronutrient availability and oxygen conditions (Zhang et al. 2018). In the marine ecosystem, microbes mediate the major redox reactions responsible for transforming energy and matter through enzymatic activity involved in key metabolic pathways (Falkowski, Fenchel, and Delong 2008). Many of those key enzymes contain/depend on micronutrients/trace metals, explaining why those metals are essential for microbial life in the ocean (Morel and Price 2003). Due to their low solubility, metal concentrations in the ocean are typically minimal, with their availability decreasing sharply within short distances from the coast (Johnson, Gordon, and Kenneth 1997). Planktonic microbes play a crucial role in the biogeochemical cycling of most essential marine metals. However, these metals also partially regulate the growth of these microbes and the microbe‐mediated cycling of carbon (C) and nitrogen (N) (Morel and Price 2003). Consequently, alterations in micronutrient availability can significantly impact microbial community structure and function (Louca et al. 2018). For example, iron (Fe) limitation has been shown to reduce the efficiency of the electron transport chain (ETC) in bacterial cells, resulting in decreased carbon‐based growth efficiency (Tortell, Maldonado, and Price 1996). Previous research has highlighted the substantial impact of micronutrient availability on primary productivity, phytoplankton community composition and carbon sequestration (Buitenhuis, Timmermans, and de Baar 2003; Saito and Goepfert 2008; Saito, Goepfert, and Ritt 2008). However, while much is known about micronutrient effects on these processes, their influence on the overall structure of marine microbial communities remains less explored.

Oxygen availability is another critical factor that shapes microbial community composition and metabolic activity (Fierer et al. 2012; Zhang et al. 2018). Microorganisms have diverse physiological adaptations to varying oxygen conditions, with aerobic, facultative anaerobic and obligate anaerobic microbes occupying distinct niches (Zhang et al. 2018). In oxygen minimum zones (OMZs), where O₂ concentrations fall below < 20 μm, micronutrients act as essential cofactors for enzymes involved in anaerobic pathways like denitrification, sulphate reduction and methane production. These processes, in turn, impact nutrient cycling and biogeochemical processes (Luther et al. 1997). The availability and cycling of micronutrients in OMZs are influenced by complex interactions between microbial activity, redox conditions and physicochemical processes. For instance, microbial‐driven denitrification and sulphate reduction affect the solubility and bioavailability of key micronutrients like Fe, Mn and Zn through redox reactions, while factors like pH, temperature and organic matter further modulate these dynamics (Luther et al. 1997). While there is substantial research on the impact of low oxygen levels on microbial communities in OMZs (Fierer et al. 2012), fewer studies have examined how micronutrients shape microbial community dynamics in such regions.

The Arabian Sea (AS) provides an ideal setting to study the intricate response of microbes to micronutrients and oxygen availability owing to its vast OMZ. The dynamic nature of the AS, influenced by upwelling, winter cooling and seasonal monsoonal winds (Madhupratap, Prasanna, et al. 1996; Prasanna et al. 2001), results in significant temporal and spatial variations in nutrient availability and primary productivity (Hansell and Peltzer 1998). The southwest monsoon enhances vertical mixing, bringing nutrient‐rich water from the bottom to the surface and increasing micro‐macronutrient concentrations (Naqvi et al. 1990). Microbial diversity has been shown to vary significantly between OMZ and non‐OMZ regions in the AS (Bandekar et al. 2018). Previous research in other marine environments has demonstrated that micronutrient availability can significantly influence the growth and activity of marine microbiomes (Tortell, Maldonado, and Price 1996), and few microbes, such as heterotrophic bacteria, have adapted to efficiently scavenge micronutrients from their surroundings by utilising high surface‐area‐to‐volume ratios and producing organic ligands to complex and transport these metals (Azam et al. 1983; Granger and Price 1999). However, despite these adaptations, there remains a gap in our understanding of the growth and activity of microbes concerning micronutrients in oxygenated versus deoxygenated waters of the AS.

For this study, we hypothesise that changes in micronutrient availability will lead to alterations in microbial community composition and diversity, with the magnitude of these effects dependent on oxygen levels. To test this hypothesis, we performed a microcosm experiment wherein we deliberately altered the micronutrient composition, including zinc (Zn), Fe, cobalt (Co) and their combination (Zn + Fe + Co) in water samples collected from oxygenated (73 m) and deoxygenated (200 m) depths of the Arabian Sea Time Series (ASTS) location. The micronutrients were chosen due to their critical roles as cofactors in microbial enzymes essential for processes such as nitrogen fixation, carbon cycling and respiration. Their availability often limits microbial growth and activity, particularly in open‐ocean environments (Morel and Price 2003). Using the 16S rRNA amplicon sequencing, we investigated the impact of micronutrient amendments on the taxonomic composition and diversity of microbial communities in the presence and absence of oxygen.

## 2 Materials and Methods

### 2.1 Microcosm Experiment

A microcosm experiment was conducted at the ASTS located at 17°0.126’N, 67°59.772′ E in the northeastern AS aboard the ORV Sindhu Sadhana in January 2020. The primary aim was to examine the impact of micronutrient modifications, encompassing Zn, Fe and Co on nutrient levels, total bacterial counts (TBCs) and microbial community structure. For this experiment, 80 L of unfiltered seawater was collected from the oxygenated (73 m) and deoxygenated (200 m) depths using a clean CTD Niskin sampler. The water sample was collected and subsequently distributed evenly among previously cleaned 5 L capacity flasks. Before the experiment, the flasks were thoroughly and sequentially cleaned with detergent, 50% v/v nitric acid (ACS grade, Sigma‐Aldrich, India) and 10% v/v hydrochloric acid (ACS grade, Sigma‐Aldrich, India). Subsequently, the flasks were retained with 10% hydrochloric acid for 1 week before being thoroughly rinsed and filled with ultrapure water (Type‐I grade) a day prior to the experiment. The ultrapure water was then discarded, and the flasks were given a final rinse with the sample seawater. Trace metals in the form of chloride salts, obtained from Sigma‐Aldrich, India, were dissolved in ACS‐grade 0.01 N HCl, with a pH of ∼1.0. After the flasks were filled with sample seawater, 1 mL of concentrated trace metal solutions, viz., Zn, Fe, Co and mix (Zn + Fe + Co), was added separately to the flasks to attain a final concentration of 10 nM L^−1^. The concentration of trace metals for the experiment was decided based on prior studies showing that this level is sufficient to trigger microbial community changes without toxicity or precipitation at higher concentrations (Hutchins et al. 2002; Eldridge et al. 2007; Becquevort, Lancelot, and Schoemann 2007). A total of 15 flasks, comprising 12 treatments (three flasks per micronutrient amendment) and 3 controls, were incubated for 5 days with exposure to a light intensity of 150 μmol m^−2^ s^−1^ under a 16:8 h light: dark cycle. To prevent any trace metal contamination, the inner walls of the incubation room were covered with thin plastic sheets prior to the experiment. Water samples were collected from control on Day 0 (C‐0) and at the end of Day 5 from control (C‐5) and treatment flasks. For nutrient analysis, 100 mL samples were collected in clean, acid‐washed bottles and preserved at −20°C until analysis (Avanzino and Kennedy 1993). TBCs were obtained by collecting 50 mL samples in sterile containers, which were fixed with buffered formaldehyde to a final concentration of 2% and stored at 4°C in the dark until analysis. For microbial community analysis, samples were filtered, and the filters were stored at −80°C until DNA extraction.

### 2.2 Physical and Chemical Parameters

For each sample, depth, temperature, salinity and pH were measured on‐site using sensors on an SBE911 CTD console mounted on an SBE 32 rosette water sampler (Sea‐Bird, Scientific). Oxygen levels were measured within a few hours of sampling using the modified Winkler titration method as described by Carpenter (1965) to ensure accurate quantification. In contrast, nutrient levels [nitrate (NO_3_^−^), nitrite (NO_2_^−^), ammonia (NH_4_^+^), silicate (SiO_3_^2−^) and phosphate (PO_4_^3−^)] were analysed from frozen samples at the National Institute of Oceanography (NIO) using a Skalar autoanalyser (Skalar Analytical, Netherlands), following established protocols to preserve sample integrity for reliable nutrient analysis (Grasshoff, Ehrhardt, and Kremling 1983).

### 2.3 Enumeration of TBCs

TBCs were assessed using a modified version of the method of Porter and Feig (1980). Aliquots of 1–5 mL from these preserved samples were subjected to incubation with 4′6‐diamidino‐2‐phenylindole (DAPI), using a working solution of 1 mg mL^−1^ (20 mL per mL of sample). Subsequently, the samples were filtered onto a 0.22 mm black polycarbonate membrane (Millipore, USA). Epifluorescence microscopy was conducted using an Olympus BX‐51 microscope, assisted by Image Pro‐plus software (Media Cybernetics, USA), and TBC was determined from a minimum of 20 randomly selected microscopic fields in each sample to ensure statistical reliability.

### 2.4 DNA Extraction and Sequencing

A sub‐sample (3.0 L) from each microcosm was filtered by passing it through a Sterivex cartridge with a 0.22 μm pore size membrane filter (Millipore, USA). This filtration process was carried out using a peristaltic pump at a flow rate of 60 mL per minute. The Sterivex cartridge was then filled with 1.8 mL of DNA storage buffer (50 mM Tris pH 8.3, 40 mM EDTA and 0.75 M sucrose), sealed and stored in a frozen state at −80°C until nucleic acid extraction following Ferrari & Hollibaugh's method (Ferrari and Hollibaugh 1999). Subsequently, the DNA was eluted and suspended in 40 mL of TE buffer. Gel electrophoresis with 0.8% agarose was performed to verify the integrity of the DNA, and the extracts were subsequently stored at −80°C for further analysis. DNA extracts were used for high‐throughput sequencing to elucidate the composition and relative abundance of bacterial communities, focusing on the V3–V4 hypervariable region of the 16S rRNA genes. This region was amplified using the primer pairs 341F (CCTAYGGGRBGCASCAG) and 806R (GGACTACNNGGGTATCTAAT). All PCR reactions were carried out with 15 μL of Phusion High‐Fidelity PCR Master Mix (New England Biolabs), 0.2 μM of forward and reverse primers and about 10 ng template DNA. Thermal cycling consisted of initial denaturation at 98°C for 1 min, followed by 30 cycles of denaturation at 98°C for 10 s, annealing at 50°C for 30 s and elongation at 72°C for 30 s and 72°C for 5 min. The 16S rRNA gene amplicons of approximately 400–450 bp were isolated using agarose gel electrophoresis (2%) and pooled at equal density ratios. Following amplification and library preparation, sequencing was performed on the paired‐end Illumina Novaseq 6000 platform by Novogene Company Limited (Cambridge, UK).

### 2.5 Post‐Sequencing Data Processing

Sequencing data processing involved several steps to ensure accuracy and reliability. Initially, paired‐end reads were demultiplexed based on unique barcodes and subsequently trimmed to remove barcode and primer sequences using trimmomatic. Paired‐end reads were merged using FLASH (V1.2.7) (Magoč and Salzberg 2011). Merged reads were further quality‐filtered using Qiime (V1.7.0) (Caporaso et al. 2010), generating a set of high‐quality clean reads. These clean reads were then compared with the SILVA reference database (V138.1), using the UCHIME algorithm (Edgar et al. 2011) to identify and subsequently eliminate chimera sequences. Operational taxonomic units (OTUs) were generated using Uparse software (v7.0.1090) (Edgar 2013) by clustering sequences with ≥ 97% similarity. Representative sequences for each OTU were selected for further taxonomic annotation. Qiime in the Mothur method was performed against the SSU rRNA database of the SILVA Database (Wang et al. 2007) for species annotation at each taxonomic rank. OTU abundance was normalised using a standard sequence number corresponding to the sample with the least sequences to remove the bias introduced by variation in the number of sequences in different samples. The 16S rRNA gene sequences were submitted to the National Centre for Biotechnology Information (NCBI) under the Bio‐Project number PRJNA1137820 with GenBank accession numbers SRX25419583‐SRX25419612.

### 2.6 Statistical Analyses

Alpha and beta diversity analyses were performed on normalised data to elucidate intra‐ and inter‐sample species diversity using Qiime. Alpha (α) diversity for each sample was assessed through six indices, including species richness, Chao1, Shannon, Simpson, abundance‐based coverage estimator (ACE) and good coverage using the vegan package in R. Non‐metric multidimensional scaling (NMDS) and analysis of similarity (ANOSIM) of total microbiota, bacteria and archaea were performed using the same package in R. Principal component analysis (PCA) was performed using the FactoMineR package and ggplot2 package in R software (V4.0.3). Using the statistical software IBM SPSS 23.0 (IBM Corp., Armonk, NY, USA), a one‐way analysis of variance (ANOVA) was conducted to understand the impact of trace metals on the TBCs and nutrients. Subsequently, a post hoc Dunnett's test was performed for comparison of the control samples Day 0 and Day 5 (#C1 and #C2) to the treated samples (#T1 (Zn), #T2 (Fe), #T3 (Co) and #T4 (Zn + Fe + Co). Canonical correspondence analysis (CCA) was carried out using Past‐3 software (https://folk.uio.no/ohammer/past/) to understand the relationship between the nutrients and microbial diversity in the control and treated samples.

## 3 Results

### 3.1 Nutrient Concentrations

In general, temperature, salinity, pH and DO were consistently higher in the oxygenated layer than in the deeper deoxygenated layer (Table S1). At a depth of 73 m, the addition of Zn, Fe, Co and their mix resulted in varied effects on nutrient concentrations compared to the control group after Day 5. Nitrate levels decreased across all treatments, except for Zn, which was comparable to the control values. Nitrite and ammonia concentrations declined in all treatments, with the lowest levels observed in the mixed treatment. Conversely, phosphate concentrations increased in all treatments, particularly in the Fe treatment. Silicate concentrations showed considerable variation, with the highest concentrations observed in the mixed treatment, significantly exceeding concentrations in other treatments (Table 1).

**TABLE 1:** Temporal variations in the major macronutrient concentration in the initial sample (Day 0) and after 5 days (Day 5) in the microcosms.

Depth	Nutrient (μmol L^−1^)	Treatment	Day 0	Day 5	Depth	Nutrient (μmol L^−1^)	Treatment	Day 0	Day 5
73 (m)	Nitrate	Con	5.74	4.74	200 (m)	Nitrate	Con	8.62	5.98
		Zn		2.86			Zn		17.39
		Fe		2.38			Fe		9.14
		Co		3.09			Co		8.27
		Zn + Fe + Co		3.21			Zn + Fe + Co		13.78
	Nitrite	Con	0.29	0.22		Nitrite	Con	0.89	0.76
		Zn		0.12			Zn		0.32
		Fe		0.13			Fe		0.63
		Co		0.13			Co		0.5
		Zn + Fe + Co		0.05			Zn + Fe + Co		0.7
	Ammonia	Con	1.65	0.26		Ammonia	Con	1.51	1.08
		Zn		1.1			Zn		1.82
		Fe		1.42			Fe		1.76
		Co		2.39			Co		1.77
		Zn + Fe + Co		0.67			Zn + Fe + Co		0.91
	Phosphate	Con	0.64	2.12		Phosphate	Con	2.91	2.12
		Zn		1.58			Zn		1.51
		Fe		2.94			Fe		0.75
		Co		0.58			Co		0.69
		Zn + Fe + Co		0.5			Zn + Fe + Co		0.94
	Silicate	Con	3.07	2.91		Silicate	Con	7.46	6.48
		Zn		2.51			Zn		9.14
		Fe		1.96			Fe		8.48
		Co		1.83			Co		6.25
		Zn + Fe + Co		16.55			Zn + Fe + Co		10.26

Abbreviations: Con = Control, Zn = Zinc, Fe = Iron, Co = Cobalt.

At a 200 m depth, nitrate increased across all treatments relative to the control with a twofold increase, particularly in the Zn and mixed treatment. Ammonia concentrations showed minimal variations across treatments. Phosphate concentrations decreased across all experimental groups compared to the control, with the lowest levels observed in the Co‐treatment. Nitrite concentrations showed variable changes across treatments but generally remained similar to the control treatment. Silicate concentrations increased in all treatment groups, with the highest levels observed in the mixed treatment (Table 1). Overall, the addition of these micronutrients altered the nutrient concentrations compared to the control group.

### 3.2 Alpha and Beta Diversity

**FIGURE 1:**
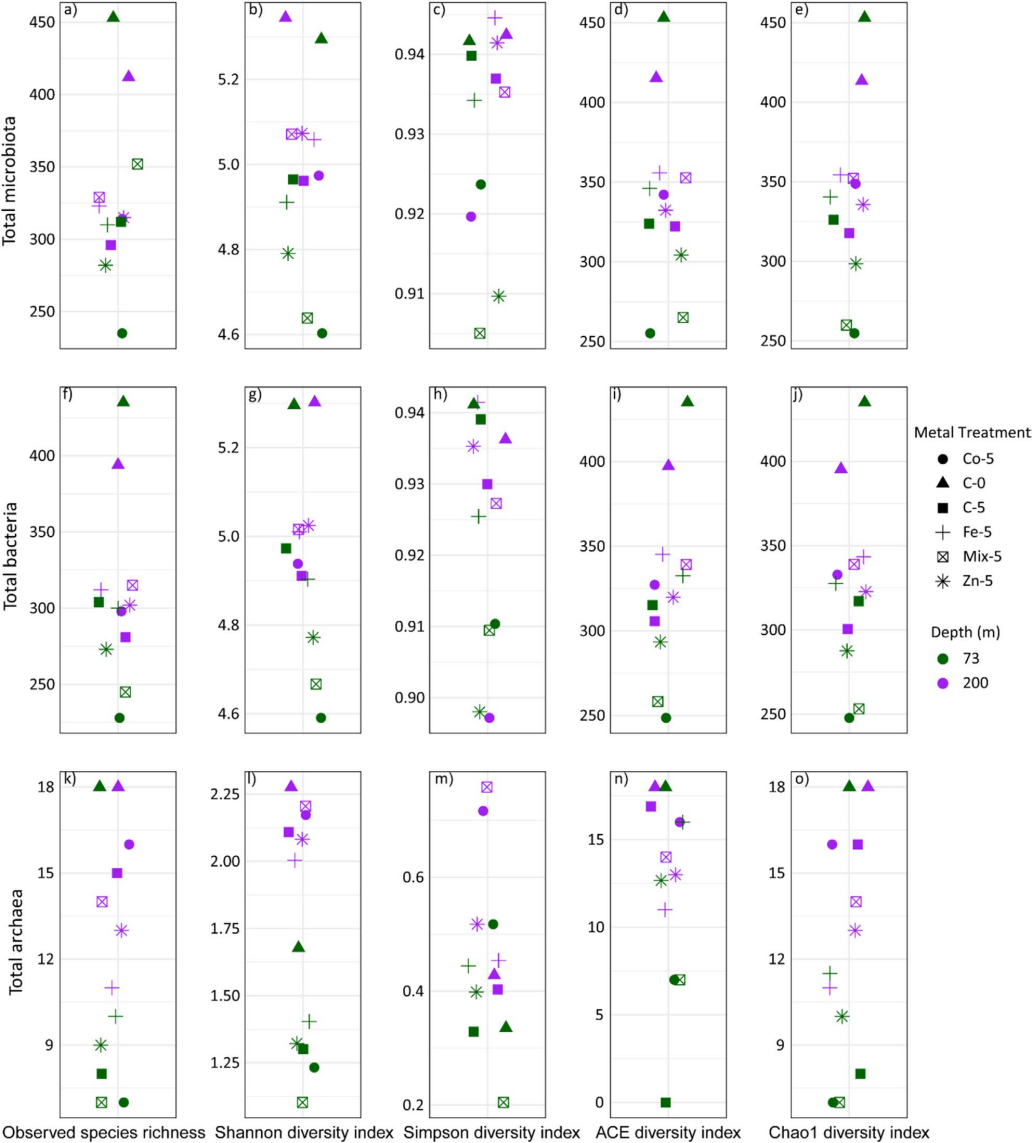
The observed species richness, Shannon, Simpson, Chao1 and ACE indices calculated for (a–e) total microbiota, (f–j) bacterial communities and (k–o) archaeal communities in the initial sample (Day 0) and after 5 days (Day 5) in the microcosms. C‐0 = Control Day 0, C‐5 = Control Day 5, Zn‐5 = Zinc Day 5, Fe‐5 = Iron Day 5, Co‐5 = Cobalt Day 5, Mix‐5 = Mixture Day 5.

Our sequence analysis indicated a near‐complete sequencing of the microbial community, with an estimated coverage of 99% (Good's coverage of 0.99) for all samples. Rarefaction curves showed saturation, suggesting deep sequencing of microbial communities (Figure S1). We evaluated the α diversity of microbial communities using diversity (Shannon, Simpson) and evenness (Chao1 and ACE) indices. Notably, C‐5 exhibited lower species richness than C‐0, although statistical analysis indicated no significant difference in the Shannon diversity. In general, at 73 m, alpha diversity indices showed minimal variation across different treatments compared to the control (Figure 1a–e). Among the treatments, Fe displayed the highest diversity (4.91), while Co (4.6) was the lowest. The mixed treatment had the highest number of OTUs (352), whereas the Co‐treatment had the lowest. This pattern was also reflected in the Chao1 and ACE indices, with the Fe treatment showing the highest estimated richness. In 200 m, the mixed treatment recorded the highest number of OTUs (329) and the highest diversity index (5.07). Overall, treated samples exhibited a higher number of OTUs than the control group (Figure 1a–e).

Separate diversity analyses for bacterial and archaeal communities revealed distinct patterns. For bacterial communities, the treated samples showed relatively stable diversity patterns at both depths. At 73 m, the number of OTUs consistently decreased in treated samples compared to the those in control, whereas at 200 m, an increase in OTUs was observed in the treated samples. The highest bacterial diversity (of 5.01) and OTU count (300) were found in Fe‐treated samples at 73 m, while the mixed samples had the highest diversity and OTUs at 200 m. Co‐treated samples recorded the lowest values in both depths (Figure 1f–j). In contrast, archaeal communities exhibited substantially lower OTUs compared to bacterial taxa at both depths. At 73 m, Fe‐treated samples had the highest OTU count (10) and diversity (1.40), while Co had the lowest. At 200 m, the Co‐treatment (16) resulted in the maximum OTUs, with the highest diversity observed in mixed samples (2.21). Fe‐treated samples had the lowest OTUs (11) and diversity at 200 m. Archaeal diversity remained relatively stable across all treatments at both depths but was higher at 200 m compared to that at 73 m (Figure 1k–o).

**FIGURE 2:**
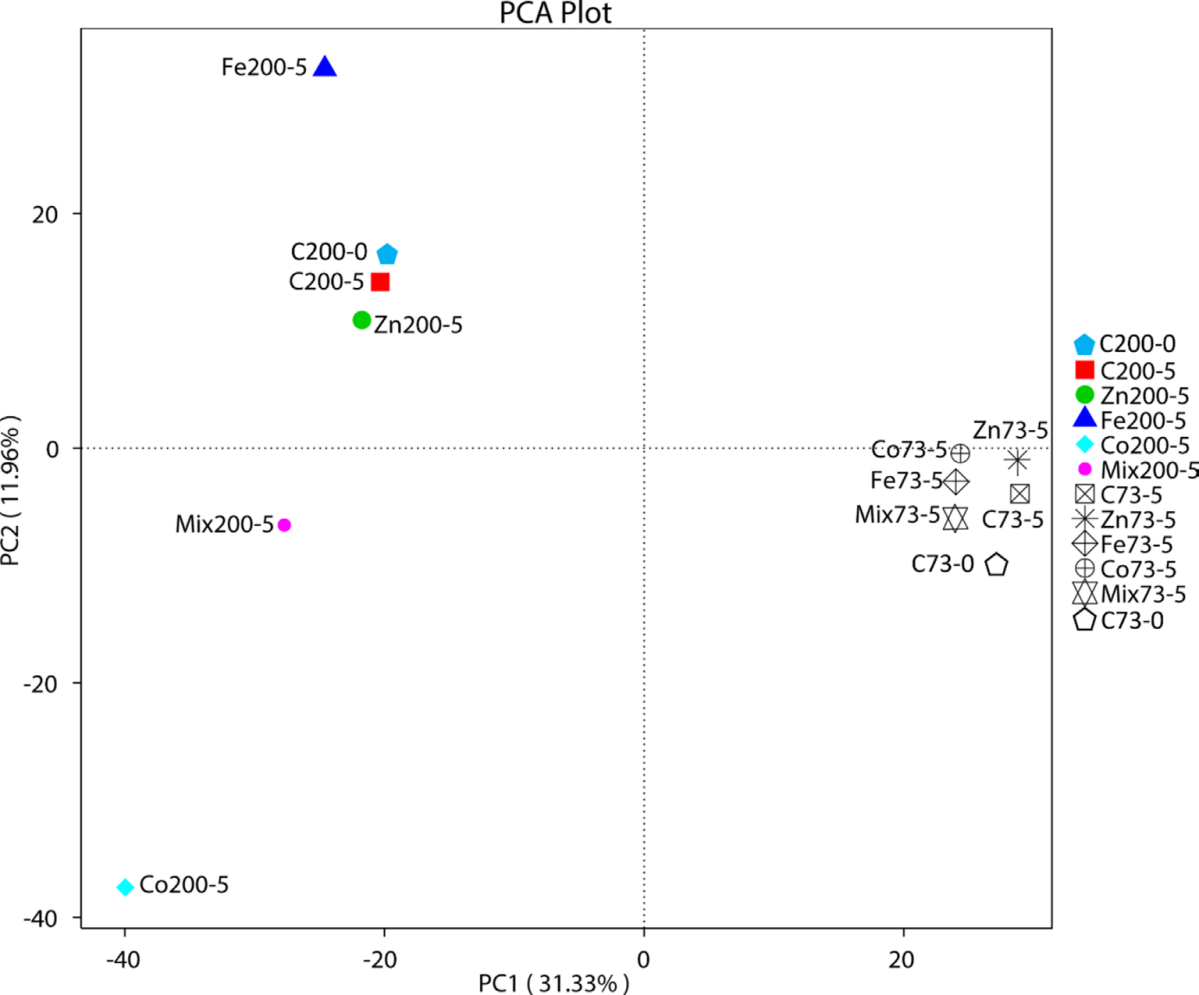
Principal component analysis (PCA) of microbial communities in the initial sample (Day 0) and after 5 days (Day 5) in the microcosms. C73‐5 = Control 73 m Day 0, Zn73‐5 = Zinc 73 m Day 5, Fe73‐5 = Iron 73 m Day 5, Co73‐5 = Cobalt 73 m Day 5, Mix73‐5 = Mixture 73 m Day 5, C200‐0 = Control 200 m Day 0, C200‐5 = Control 200 m Day 5, Zn200‐5 = Zinc 200 m Day 5, Fe200‐5 = Iron 200 m Day 5, Co200‐5 = Cobalt 200 m Day 5, Mix200‐5 = Mixture 200 m Day 5.

**FIGURE 3:**
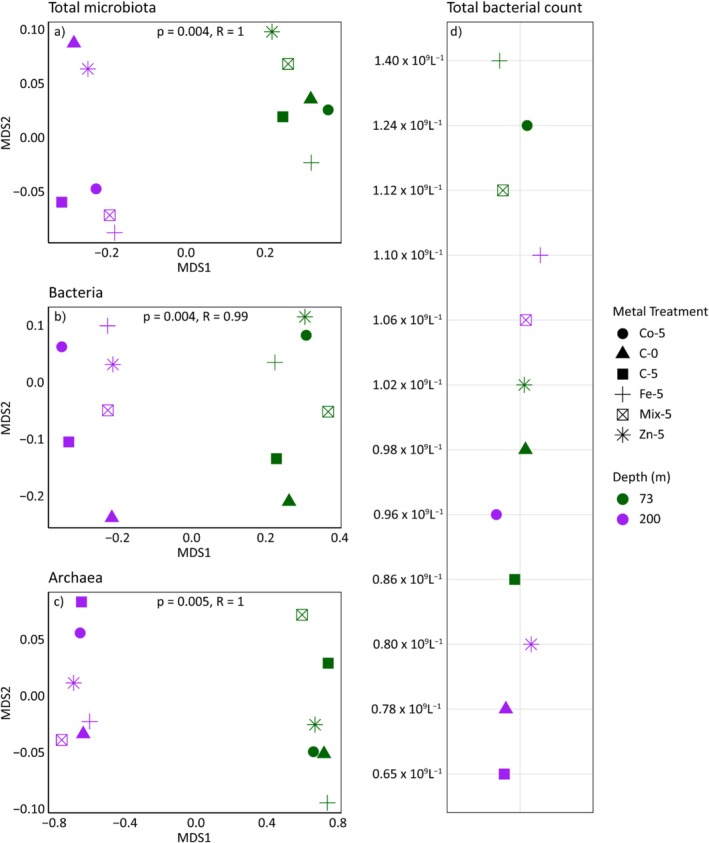
Non‐metric multidimensional scaling (NMDS) analysis of (a) total microbiota, (b) bacterial communities, (c) archaeal communities and (d) total microbial counts in both the initial sample (Day 0) and after 5 days (Day 5) in the microcosms. See Figure 1 for abbreviations.

The effects of micronutrient treatments on microbial communities at 73 m and 200 m depths are demonstrated using the PCA plot (Figure 2). PC1 and PC2 together capture a significant portion accounting for 31.33% and 11.96%, respectively. Samples from 200 m, C200‐0 and C200‐5 (control samples) indicated a similar community structure between Day 0 and Day 5 controls. However, the trace metal treatments like Fe200‐5, mix200‐5 and especially Co200‐5 showed significant deviations along PC1 and PC2, reflecting distinct community shifts due to these treatments. For the 73 m depth, the control samples (C73‐0 and C73‐5) are tightly clustered, with moderate shifts in treated samples, particularly for Zn and mix treatments. Overall, each treatment results in distinct bacterial community structures compared to the controls, indicating that these micronutrients alter bacterial communities. The clear separation of samples from 73 to 200 m along the PCA axes highlights the depth‐dependent effects of these micronutrient treatments, with all treatments generally having a positive effect on the bacterial communities. Overall, the NMDS (Figure 3a) plot shows a very low‐stress value (0.001), indicating an excellent representation of the microbial community in two dimensions. The NMDS analysis reveals depth‐dependent separation of bacterial (Figure 3b) and archaeal (Figure 3c) diversity across all micronutrient treatments.

### 3.3 TBC

The TBCs at both depths, 73 and 200 m (Figure 3d), show distinct responses to micronutrient amendments. At both depths, higher bacterial counts were observed in all treatments compared to their control groups. At 73 m, the highest bacterial count was observed in the Fe‐enriched flask (1.4 × 10^9^ L^−1^), followed by Co (1.24 × 10^9^ L^−1^), mix (1.12 × 10^9^ L^−1^) and Zn (1.02 × 10^9^ L^−1^), all exceeding the control groups 0 (0.98 × 10^9^ L^−1^) and 5 (0.86 × 10^9^ L^−1^). At 200 m, Fe treatment again showed the highest TBC (1.1 × 10^9^ L^−1^), followed by the mixed treatment (1.06 × 10^9^ L^−1^) and Co (0.96 × 10^9^ L^−1^). The Zn‐amended flasks showed a smaller increase (0.8 × 10^9^ L^−1^) compared to the control groups 0 (0.78 × 10^9^ L^−1^) and 5 (0.65 × 10^9^ L^−1^). Overall, bacterial counts were consistently higher at 73 m compared to those at 200 m, with Fe treatment showing the most pronounced effect at both depths.

### 3.4 Prokaryotic Community Structure and Abundance

The taxonomic composition of all samples was evaluated by analysing sequences with an abundance of ≥ 1%, representing the prokaryotic community. The relative abundance analysis focused on the top 10 most abundant genera in each treatment. Initially, the sequences were examined at the phylum level, followed by a more detailed analysis at the genus level for enhanced resolution. The biological replicates showed high consistency and were statistically comparable. Therefore, we pooled the data from the replicates for each treatment into a single representation for community data analysis.

#### 3.4.1 Bacterial and Archaeal Communities in Oxygenated Layer

At an oxygenated depth of 73 m, *Proteobacteria* dominated all treatments, comprising 42%–52% of the community, with the highest abundance (52%) observed in the Fe treatment (Table S2). *Actinobacteriota* (20.9%) and *Thermoplasmatota* (19.2%) were the next most abundant phyla in the control groups. In the Zn and Co treatments, *Actinobacteriota* decreased in abundance relative to *Bdellovibrionota*, which increased to 22.5% and 12%, respectively. Conversely, *Thermoplasmatota* consistently decreased across all treatments, except for a significant increase in the mixed treatment (19.9%). *Cyanobacteria* exhibited a substantial decline across treatments (1.5%–3.1%) compared to the control sample (8%) (Table S2).

At the genus level, the bacterial abundance varied distinctly among metal treatments (Table 2). In the control, *Clade_Ia* (15.8%) and *Candidatus_Actinomarina* (10.6%) were the most abundant, alongside notable amounts of unclassified bacteria (23.7%). In the Zn treatment, *Peredibacter* increased significantly to 23.2%, while *Clade_Ia* (15.8%) and *Candidatus_Actinomarina* (7.2%) remained prominent. The Fe treatment showed a similar distribution to the control, while the Co treatment saw a slight increase in *Clade_Ia* (21%) and *Candidatus_Actinomarina* (12.5%), alongside a notable increase in *Peredibacter* (12%). The mixed metal treatment displayed a varied distribution, with significant contributions from *Candidatus_Actinomarina* (18.5%) and *Clade_Ia* (17.7%). Across all treatments, unclassified bacteria (17.5%–23.8%), *Clade_Ia* (15.6%–21.07%) and *Candidatus_Actinomarina* (7.1%–18.45%) consistently showed high abundance, while *Peredibacter* notably increased in Zn and Co treatments. The archaeal community analysis (Table 3) showed that *Marine Group II* (46%–88%) was predominant, although it declined in Fe (69%) and Co (46%) treatments compared to the control (79%). *Candidatus Nitrosopelagicus* showed a moderate increase in Zn (24.5%) and Fe (27%) treatments, with a substantial increase in the Co treatment (51%) relative to the control (18.1%). Other taxa including *Woesearchaeales*, *Nitrosopumilaceae*, *Marine Group III*, *Marine Benthic Group A*, *SCGC AAA011‐D5* and *Candidatus Iainarchaeum* were present in smaller proportions.

**TABLE 2:** Relative abundance percentages (*x*‐axis) and depthwise distribution (*y*‐axis) of top 10 bacterial genera in the initial sample (Day 0) and after 5 days (Day 5) in the microcosms.

	73 m						200 m					
Bacterial genera	C73‐0	C73‐5	Zn73‐5	Fe73‐5	Co73‐5	Mix73‐5	C200‐0	C200‐5	Zn200‐5	Fe200‐5	Co200‐5	Mix200‐5
Unclassified bacteria	24.98	23.72	19.11	21.61	18.91	17.48	22.52	19.32	19.81	19.98	16.61	19.16
*Marinimicrobia_*(*SAR406_clade*)	2.96	3.01	1.82	4.10	0.87	5.10	5.96	6.46	6.54	8.91	25.42	16.04
*Sva0996_marine_group*	3.66	3.73	2.12	5.33	2.00	2.81	7.53	8.16	5.97	9.87	11.62	7.95
*SUP05_cluster*	0.00	0.00	0.00	0.00	0.00	0.00	6.64	6.84	6.23	9.72	9.89	10.41
*LWQ8*	0.00	0.00	0.00	0.00	0.00	0.00	0.16	0.18	16.37	1.97	0.11	13.52
*UBA10353_marine_group*	0.00	0.00	0.00	0.00	0.00	0.00	7.76	7.46	4.85	5.90	4.51	5.09
*Alcanivorax*	0.00	0.00	0.00	0.00	0.00	0.00	16.25	17.37	1.04	2.21	2.90	4.22
*SAR324_clade* (*Marine_group_B*)	4.39	4.47	4.46	8.94	3.54	6.59	8.52	8.66	2.81	4.57	4.17	3.59
*Erythrobacter*	1.05	1.06	8.89	0.99	1.84	0.68	0.85	1.12	7.06	11.97	1.35	2.02
*Nocardioides*	4.46	4.54	0.32	0.30	0.06	0.18	0.57	0.52	10.69	7.01	0.33	3.90
*Clade_Ia*	15.49	15.78	15.76	15.62	21.07	17.74	4.60	5.29	4.27	3.06	2.47	2.54
*SAR202_clade*	0.68	0.68	0.59	0.77	0.73	2.86	2.62	2.72	2.12	1.59	6.57	3.31
*Clade_Ib*	2.40	2.47	3.67	4.33	4.88	5.17	5.51	5.83	3.17	2.81	1.49	1.80
*AEGEAN‐169_marine_group*	2.65	2.70	4.61	3.40	4.70	6.42	4.38	4.22	2.16	3.45	1.22	1.69
*Alteromonas*	0.00	0.00	0.00	0.00	0.00	0.00	0.93	0.76	4.11	3.78	1.72	1.65
*Clade_II*	6.89	7.02	4.95	8.51	6.70	8.20	3.70	3.62	2.25	1.49	1.62	1.30
*Peredibacter*	0.29	0.29	23.21	0.67	12.02	0.37	0.64	0.72	0.41	1.07	5.34	0.55
*Ketobacter*	0.00	0.00	0.00	0.00	0.00	0.00	0.77	0.74	0.16	0.63	2.66	1.26
*Pseudomonas*	0.04	0.03	0.01	0.09	2.47	0.18	0.00	0.00	0.00	0.00	0.00	0.00
*DEV007*	0.62	0.63	0.07	0.36	4.01	0.18	0.00	0.00	0.00	0.00	0.00	0.00
*SAR86_clade*	2.45	2.49	1.72	3.07	1.22	2.59	0.00	0.00	0.00	0.00	0.00	0.00
*Cyanobacteriaceae*	6.84	6.95	0.92	0.77	2.13	2.57	0.00	0.00	0.00	0.00	0.00	0.00
*Oleibacter*	4.33	4.41	0.54	6.91	0.38	2.09	0.00	0.00	0.00	0.00	0.00	0.00
*Candidatus_Actinomarina*	10.39	10.59	7.19	14.17	12.45	18.45	0.00	0.00	0.00	0.00	0.00	0.00
*Mycobacterium*	5.33	5.43	0.05	0.07	0.02	0.33	0.00	0.00	0.00	0.00	0.00	0.00

*Note:* See Figure 2 for abbreviations.

**TABLE 3:** Relative abundance percentages (*x*‐axis) and depthwise distribution (*y*‐axis) of top 10 archaeal genera in the initial sample (Day 0) and after 5 days (Day 5) in the microcosms.

	73 m						200 m					
Archaeal genera	C73‐0	C73‐5	Zn73‐5	Fe73‐5	Co73‐5	Mix73‐5	C200‐0	C200‐5	Zn200‐5	Fe200‐5	Co200‐5	Mix200‐5
*Nitrosopumilaceae*	0.39	0.33	0.33	0.41	0.18	0.03	74.74	76.54	67.73	72.77	17.95	32.66
*Marine_Benthic_Group_A*	0.16	0.12	0.04	0.02	0.00	0.02	8.57	8.03	7.63	7.17	5.83	8.93
*Woesearchaeales*	0.61	0.55	0.25	0.25	0.03	0.17	6.46	5.65	12.16	9.53	42.91	30.65
*Candidatus_Iainarchaeum*	0.04	0.01	0.01	0.01	0.00	0.01	2.59	2.46	1.59	1.00	1.43	1.61
*Candidatus_Nitrosopelagicus*	18.15	18.22	24.55	27.97	51.70	8.92	1.50	2.19	2.38	3.91	0.46	1.46
*SCGC_AAA011‐D5*	0.04	0.00	0.01	0.01	0.00	0.01	1.69	1.67	3.44	0.85	2.95	2.83
*Marine_Group_III*	0.76	0.71	0.22	1.65	0.99	2.06	1.76	1.57	3.34	2.31	24.84	17.49
*Unclassified bacteria*	0.35	0.19	1.02	0.61	0.73	0.10	2.06	1.08	0.71	1.45	0.64	1.04
*Marine_Group_II*	79.46	79.86	73.55	69.07	46.37	88.71	0.60	0.81	1.04	1.00	2.98	3.32

*Note:* See Figure 2 for abbreviations.

#### 3.4.2 Bacterial and Archaeal Communities in Deoxygenated Layer

In the deeper deoxygenated depth of 200 m, *Proteobacteria* was the predominant phylum across all samples, exhibiting a consistent decrease in relative abundance, particularly in the Co (43.9%) and mixed (48.9%) treatments. Conversely, the relative abundance of *Actinobacteriota* increased compared to that of the control, with significant increases in the Zn (16.9%) and Fe (18.9%) treatments. *SAR324 clade* decreased across all treatments, while *Marinimicrobia* increased significantly in the Co (22.9%) and mixed (15.3%) treatments, surpassing control values. The abundance of *Patescibacteria* increased notably in the Zn (13.9%) and mixed (12.9%) treatments compared to that of control (0.97%). In contrast, *Crenarchaeota* consistently declined in relative abundance across treatments, and *Nanoarchaeota* showed a notable increase in the Co‐treated (8.9%) samples (Table S2).

The genus‐level analysis at 200 m revealed distinct bacterial compositions across treatments (Table 2). In the control sample, *Alcanivorax* was dominant (17.4%), followed by unclassified bacteria (19.3%), *SAR324_clade* (8.7%), *Sva0996_marine_group* (8.6%), *UBA10353_marine_group* (7.5%), *SUP05_cluster* (6.8%) and *Marinimicrobia* (6.5%). The Zn treatment showed significant increases in *LWQ8* (16.4%), *Nocardioides* (10.7%) and *Erythrobacter* (7%). The Fe treatment exhibited similar patterns to the Zn treatment, while the Co treatment was marked by significant increases in *Marinimicrobia* (25.4%) and *Sva0996_marine_group* (11.6%). Across all treatments, *Marinimicrobia*, *SUP05_cluster*, *Sva0996_marine_group*, *UBA10353_marine_group* and unclassified bacteria remained consistently abundant. *Alteromonas* was more prevalent in the Zn (4.1%) and Fe (3.8%) treatments, while *Peredibacter* (5.3%) and *Ketobacter* (2.7%) increased notably in the Co treatment. In the archaeal community (Table 3), *Nitrosopumilaceae* dominated in the control sample (76.5%). The Zn (67.7%) and Fe (72.7%) treatments closely resembled this pattern, but the Co (17.9%) and mixed (32.6%) treatments resulted in a significant shift, with *Woesearchaeales* increasing substantially (30%–42%) while *Nitrosopumilaceae* decreased (17%–32%). Additionally, *Marine Group III* saw a notable increase (17%–24%) in both Co and mixed treatments.

### 3.5 Effect of Environmental Parameters on Bacterial and Archaeal Community Structure

The ANOVA results show highly significant differences in all measured parameters (nitrate, nitrite, ammonia, phosphate, silicate and TBC) across depths (Table 4), as indicated by the extremely high *F*‐values and *p*‐values < 0.0001 at both 73 and 200 m. These results suggest strong variability in nutrient concentrations and microbial activity between these depths indicating significant nutrient stratification in the water column. Similarly, high *F*‐values for TBC demonstrate strong depth‐related variations in biogeochemical processes. The post hoc Dunnett's multiple comparison test (Table 5) further confirms the significant findings from the one‐way ANOVA results. The comparisons between control Day 0 and various treatments show that treatments significantly (*p* < 0.0001) impact TBC and nutrients at both depths. In contrast, the control day 5 did not show significant changes compared to control day 0, confirming stability over time without treatment.

**TABLE 4:** One‐way ANOVA analysis and significance (*p* < 0.05) of variations in total bacterial counts and nutrients in samples amended with metals (Zn, Fe, Co, and Zn + Fe + Co).

	73 m		200 m	
Parameters	*F*	*p*	*F*	*p*
Nitrate	8177.978	< 0.0001	515,881	< 0.0001
Nitrite	295.7	< 0.0001	53.988	< 0.0001
Ammonia	578.497	< 0.0001	3355.2	< 0.0001
Phosphate	1644.526	< 0.0001	32546.9	< 0.0001
Silicate	996,060.5	< 0.0001	94970.9	< 0.0001
TBC	1161.6	< 0.0001	977.3	< 0.0001

**TABLE 5:** Post hoc Dunnett's multiple comparison (two‐sided) for total bacterial counts and metal treatments (Zn, Fe, Co and Zn + Fe + Co).

Dependent variable	(I) Treatments	(J) Treatments	*p* (73 m)	*p* (200 m)
	#C2	#C1	1	1
	#T1		< 0.0001	< 0.0001
Nitrate	#T2		< 0.0001	< 0.0001
	#T3		< 0.0001	< 0.0001
	#T4		< 0.0001	< 0.0001
	#C2	#C1	0.109	1
	#T1		< 0.0001	0.001
Nitrite	#T2		< 0.0001	0.001
	#T3		< 0.0001	< 0.0001
	#T4		< 0.0001	0.361
	#C2	#C1	0.963	1
	#T1		< 0.0001	< 0.0001
Ammonia	#T2		< 0.0001	< 0.0001
	#T3		< 0.0001	< 0.0001
	#T4		< 0.0001	< 0.0001
	#C2	#C1	0.963	1
	#T1		< 0.0001	< 0.0001
Phosphate	#T2		< 0.0001	< 0.0001
	#T3		0.324	< 0.0001
	#T4		< 0.0001	< 0.0001
	#C2	#C1	0.109	1
	#T1		< 0.0001	< 0.0001
Silicate	#T2		< 0.0001	< 0.0001
	#T3		< 0.0001	< 0.0001
	#T4		< 0.0001	< 0.0001
	#C2	#C1	< 0.0001	<0.0001
	#T1		< 0.0001	< 0.0001
TBC	#T2		< 0.0001	0.013
	#T3		< 0.0001	< 0.0001
	#T4		< 0.0001	< 0.0001

*Note:* The control Day 0 (#C1) was compared with the control Day 5 (#C2), Zn (#T1), Fe (#T2), Co (#T3) and Zn + Fe + Co (#T4) treatments. The mean difference is significant at *p* < 0.05 level.

**FIGURE 4:**
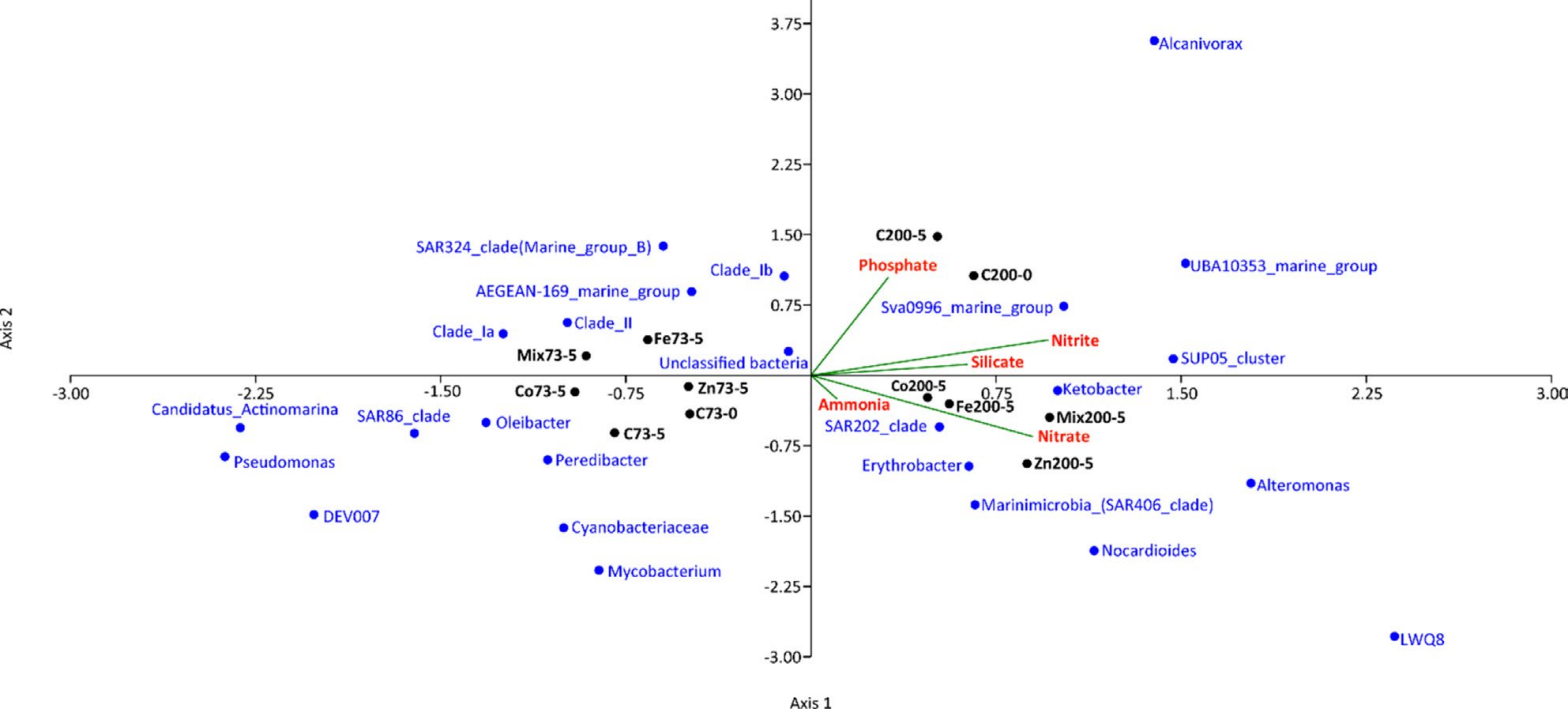
Canonical correspondence analysis (CCA) ordination plot of bacterial communities associated with environmental variables from oxygenated (73 m) and deoxygenated (200 m) depths of the Arabian Sea Time Series (ASTS) location. Red is environmental parameters, black is treatment and depth and blue is bacterial community. See Figure 2 for abbreviations.

The CCA ordination plot (Figure 4) illustrated the significant impact of environmental variables and micronutrients on bacterial community structure at both depths. The analysis revealed a clear separation and distinct clustering of bacterial families between oxic and suboxic zones. Bacterial groups such as *Sava0996_marine_group*, *SUP05_cluster*, *Alcanivorax* and *UBA10353_marine_group* were positively correlated, indicating their proliferation in environments rich in phosphate, silicate and nitrite, in contrast to groups like *Candidatus_Actinomarina* and *Pseudomonas*. Additionally, clusters including *SAR324_clade* (*Marine_group_B*), *AEGEAN‐169_marine_group*, *Clade_Ia*, *Clade_Ib* and *Clade_II* were primarily influenced by Fe and mixed treatments. Other bacterial taxa, such as *Ketobacter*, *Erythrobacter*, *SAR202_clade*, *Marinimicrobia* (*SAR406_clade*), *Alteromonas*, *Nocardioides* and *LWQ8*, showed positive correlations with ammonia, nitrate Co, Fe, Zn and mixed treatments.

**FIGURE 5:**
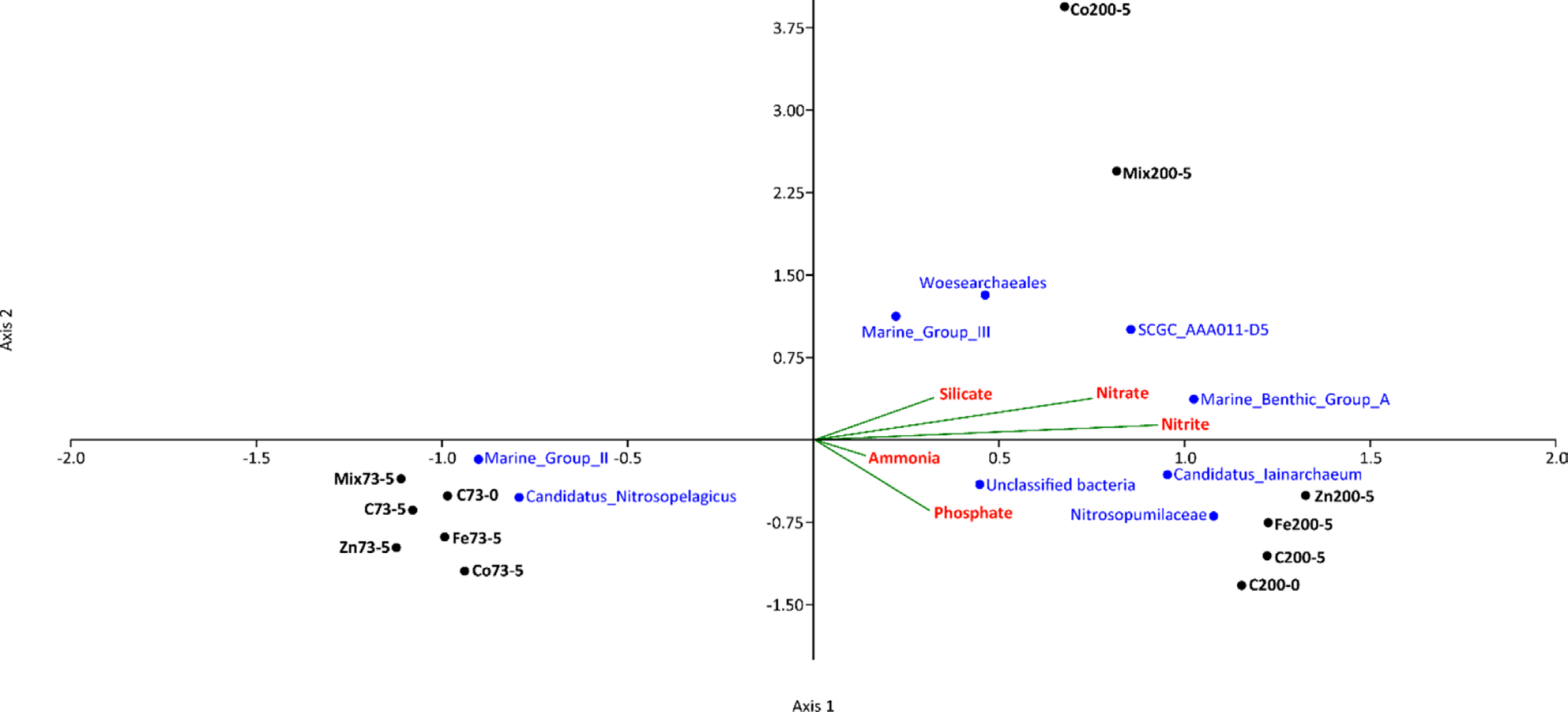
Canonical correspondence analysis (CCA) ordination plot of archaeal communities associated with environmental variables from oxygenated (73 m) and deoxygenated (200 m) depths of the Arabian Sea Time Series (ASTS) location. Red is environmental parameters, black is treatment and depth and blue is archaeal community. See Figure 2 for abbreviations.

Similarly, the archaeal CCA plot (Figure 5) showed that Axis 1 accounted for the majority of variance. Taxa such as *Marine_Benthic_Group_A*, *CSGC_AAA011‐D5*, *Woesearchaeles*, *Marine_Group_III*, *Candidatus_lainarchaeum*, *Nitrosumpimilaceae* and unclassified archaea showed strong positive associations with nitrate, nitrite and phosphate concentrations. In contrast, *Marine_Group_II* and *Candidatus_Nitrosopelagicus* were negatively correlated with these nutrients, indicating an inverse relationship. Additionally, *Woesearchaeales* and *Marine_Group_III* were positively associated with higher silicate levels. Control (C73‐0, C73‐5) and treated (Fe73‐5, Zn73‐5, Co73‐5) samples exhibited minimal influence on archaeal diversity. However, control samples (C200‐0, C200‐5) and treated samples (Fe200‐5 and Zn200‐5) shift towards the positive end of Axis 1 on Day 5, highlighting a significant influence of Fe and Zn on *Candidatus_lainarchaeum*, *Nitrosumpimilaceae*and unclassified archaea.

## 4 Discussion

The role of micronutrients as primary regulators of microbial communities is gaining increased recognition within marine ecosystems (Bertrand et al. 2015). Micronutrients are involved in secondary metabolism and redox reactions, with their availability primarily influencing microbial community assembly (Dai et al. 2023). Prior research has demonstrated that micronutrient levels impact heterotrophic microbial activity and diversity in freshwater as well as global oceans (Ramaiah et al. 2015; Jain et al. 2015; Baltar et al. 2018). Nonetheless, the contribution of micronutrients to the structure of microbiomes in oxygen‐deficient regions remains largely unexplored. Our findings, based on the microcosm experiment, highlight the significance of micronutrients in shaping microbial community composition within the AS‐OMZ. Additionally, we investigate the effect of micronutrients on microbial diversity in both the oxygenated surface waters and the deep deoxygenated waters of the AS‐OMZ.

### 4.1 Effect on Nutrient Concentration

Our observation of reduced nitrate and silicate concentrations in treatments from the upper oxygenated layer aligns with findings from the Eastern Tropical Pacific, where individual Fe and Zn treatments influence nitrate and silicate levels (Franck et al. 2003). Moreover, Fe availability is known to limit nitrate‐reducing enzymes (Morel and Price 2003). Thus, Fe addition may have facilitated nitrate reduction, leading to its decreased concentration. Conversely, Zn is reported to inhibit nitrate reduction (Waara 1992; Stone et al. 2006), yet our findings show nitrate utilisation, suggesting the need for further investigation into the biochemical pathways of bacterial nitrate reduction. Moreover, the significant increase in silicate concentration in mixed treatment implies that the Fe, Zn and Co can collectively stimulate the growth and activity of heterotrophic bacteria and zooplankton that grazes on the diatoms, thereby releasing silicate into the water column (Brzezinski, Villareal, and Lipschultz 1998; Shaked and Lis 2012).

The elevated nitrate and silicate concentrations at deeper deoxygenated depths indicate microbially mediated recycling of organic matter, nitrogen and decomposing diatoms from the initial sample (Bidle and Azam 2001; Sanders et al. 2007). In the microcosm, these higher concentrations compared to the control suggest rapid mineralisation of organic matter and nutrient regeneration via the microbial loop (Becquevort, Lancelot, and Schoemann 2007). The remarkably high nitrate levels in the treated flasks may result from microbial processes such as anammox or coupled nitrification–denitrification. In this controlled environment, enhanced nitrification drives ammonia oxidation to nitrate, while denitrification may not sufficiently remove it. This suggests that oxygen in the upper layers promotes efficient recycling of organic matter and phytoplankton growth, whereas deeper deoxygenated layers inhibit these processes, leading to nutrient accumulation and highlighting oxygen's critical role in regulating nutrient dynamics within the AS‐OMZ.

### 4.2 Effect on TBCs

The increased bacterial counts within Fe, Zn, Co and mixed microcosms following incubation suggest that bacterial growth in the AS‐OMZ may be limited by Fe, Zn and Co and co‐limited by all three. These elements may either directly or indirectly affect microbial metabolism or induce changes in the quality of organic matter produced by phytoplankton or both. Previous studies have demonstrated that Fe addition directly stimulates bacterial growth, as observed in microcosm experiments in the Eastern Equatorial Pacific Ocean (Cochlan 2001) and the California Current System (Manck et al. 2024). The direct impact of Co addition on marine heterotrophic bacterial growth or abundance remains unclear (Antony et al. 2011), except for certain marine *Cyanobacteria*, including members of the genera *Synechococcus* and *Prochlorococcus*, which specifically require Co for growth (Sunda and Huntsman 1995; Saito et al. 2002). In contrast, the effect of Zn addition on marine heterotrophic bacteria is reported to be concentration‐dependent with minimal impact on bacterial abundance (Bong et al. 2010, Rasmussen and Olapade 2016), consistent with our findings.

### 4.3 Effect on Microbial Diversity and Evenness

The observed decrease in microbial diversity and evenness indices following incubation in the upper layer suggests an enrichment of specific phylotypes. Microbial diversity in aquatic systems is governed by both bottom‐up and top‐down factors with resource competition and access to bioavailable organic carbon often limiting microbial growth (Kirchman and Rich 1997; Van Wambeke et al. 2002). The reduced diversity observed in our study likely results from bottom‐up controls. However, since unfiltered seawater was used in the experiment, the potential top‐down influence on diversity cannot be ruled out.

In the oxygenated layer, the highest diversity of both bacteria and archaea was observed in the Fe treatment. Conversely, in the deoxygenated layer, the mixed treatment exhibited the maximum diversity. This can be attributed to Fe being a crucial limiting nutrient in oxygenated waters, which stimulates phytoplankton growth and subsequently supports a diverse array of heterotrophic bacteria and archaea (Moore et al. 2013). In deoxygenated waters, the microbial community is dominated by anaerobic or facultatively anaerobic microorganisms that require a combination of micronutrients to sustain their metabolic processes (Twining and Baines 2013). For example, Zn is critical for dehydrogenases involved in fermentation; Fe is essential for nitrogenase, an enzyme involved in nitrogen fixation (Twining and Baines 2013); and Co is necessary for the synthesis of vitamin B12, which is required for enzymes involved in anaerobic metabolism, such as methylmalonyl‐CoA mutase and methionine synthase (Saito, Rocap, and Moffett 2005; Noble et al. 2008). Therefore, the presence of these micronutrients fosters greater diversity among anaerobic microorganisms. These findings highlight the critical role of oxygen availability in influencing nutrient dynamics and microbial community composition in marine ecosystems.

### 4.4 Effect on Prokaryotic Community Structure

The taxonomic analysis of microbial communities at the phylum and genus levels revealed distinct responses to micronutrient amendments in both oxygenated and deoxygenated depths, emphasising the critical influence of oxygen availability and the diverse reactions to various micronutrients. The microbial community in the water samples collected from the ASTS for the microcosm experiment was predominantly composed of *Proteobacteria*, *Actinobacteria* and *Thermoplasmatota* (formerly *Marine_Group_II*). These observations align with prior findings from the AS‐OMZ (Bandekar et al. 2018; Fernandes, Shenoy, and Damare 2020; Paingankar et al. 2020; Li et al. 2024).

#### 4.4.1 Phylum‐Level Abundance

In aerobic environments, *Proteobacteria* demonstrate significant metabolic flexibility, utilising various metal resistance mechanisms such as efflux pumps and sequestration to withstand metal exposure (Nies 2003; Emerson, Fleming, and McBeth 2010). However, under anaerobic conditions, shifts in metal bioavailability and toxicity can inhibit *Proteobacteria* reliant on aerobic respiration (Gadd 1992, 2010), leading to population declines. Conversely, *Actinobacteria*, prevalent in these layers, play a pivotal role in organic matter decomposition but may face reduced abundance under metal stress due to disruptions in enzymatic processes (Haferburg and Kothe 2007). In deoxygenated waters, metals like Fe and Zn may act as cofactors in metabolic processes, potentially enhancing their abundance as observed in our study (Haferburg and Kothe 2007). The exclusive presence of *Thermoplasmatota* in oxygenated water samples and the significant increase in mixed treatments suggests a synergistic effect, activating diverse metabolic pathways to cope with stress (Distaso et al. 2020; Yang et al. 2022). Similarly, the elevated abundance of *Bdellovibrionota* in Zn and Co treatments may be attributed to their predatory behaviour, advantageous for nutrient acquisition under stressed conditions (Sockett 2009). Conversely, the pronounced decline of *Cyanobacteria* across all treatments highlights their sensitivity to metal toxicity, impairing their photosynthetic machinery (Cassier‐Chauvat and Chauvat 2014).

#### 4.4.2 Genus‐Level Abundance

In the oxygenated layer, *Clade_Ia* bacteria increase in response to Co, indicating adaptability to this metal (Dulaquais et al. 2014). Similarly, *Candidatus_Actinomarina* thrives under Fe, Co and mixed treatments, suggesting that these elements are key metabolic cofactors (Genchi et al. 2023). *Peredibacter* increases in Zn and Fe exposures, possibly due to enhanced metabolic processes (Simon, Robinson, and Rodríguez‐Quiñones 2003; Saper and Rash 2009). The proliferation of *SAR324* in Fe treatments suggests involvement in Fe‐dependent oxidation and reduction, although its role remains unclear (Boeuf et al. 2021; Baltar et al. 2018). The increase in *AEGEAN‐169* and *Clade Ib* across treatments reflects their capacity to use micronutrients, providing a competitive advantage in metal‐rich environments (Coclet et al. 2019). The decline in *Mycobacterium* and *Nocardioides* populations may result from metal toxicity, leading to genetic changes and disease susceptibility (Nies 2003; Xiong and Yuan 2019). The prevalence of unclassified bacteria indicates novel communities adapting to higher micronutrient levels (Coclet et al. 2019). In archaeal communities, *Marine Group II* (MG‐II) predominates surface waters, due to the metal toxicity resistance mechanism (Timkov et al. 2019). *Candidatus Nitrosopelagicus*, a marine ammonia‐oxidising archaeon, increases with Co, likely due to its ability to produce cobalamin, an essential metabolic cofactor (Santoro et al. 2015). Higher Co levels enhance cobalamin production, promoting *Nitrosopelagicus* growth, while its stability in other treatments may result from adaptation via metal resistance genes or inherent tolerance to elevated metal concentrations (Steinert et al. 2020).

In deoxygenated environments, bacterial genera, *Marinimicrobia* (*SAR406 clade*) and *SUP05*, use sulphur and nitrogen as electron acceptors (Bertagnolli et al. 2017; Rathore, Sheikh, and Singh 2021). These metals enhance enzymes like Fe/Co‐dependent nitrile hydratase and polysulphide reductase important for nitrogen and sulphur cycling (Dulaquais et al. 2014; Hawley et al. 2017). LWQ8 increases with Zn and mixed treatments, highlighting Zn′s role as a metalloenzyme cofactor *Alcanivorax*, involved in hydrocarbon degradation, declines in metal treatments, possibly due to enzyme inhibition or altered in metal‐ion‐driven pathways (Zadjelovic et al. 2020; Sirajuddin et al. 2014; Coclet et al. 2019). *SAR202*, common in metal‐rich deep waters, likely oxidises recalcitrant organic matter, and more research is needed to confirm if Co enhances its oxidation capacity in low‐oxygen environments (Landry et al. 2017). *Erythrobacter*, an aerobic photoheterotroph, increases in Zn‐ and Fe‐treated deoxygenated waters, suggesting that these metals promote alternative metabolic pathways in oxygen‐deficient environments. In contrast, *SAR324*, *Clade_Ia*, *Clade_Ib*, *AEGEAN‐169* and *UBA10353* decline in anaerobic environments, likely due to limited metabolic versatility (Chen et al. 2021). Archaeon, *Nitrosopumilaceae*, linked to ammonia oxidation, predominates in the control, Zn and Fe treatments but reduces with Co. The mixed treatment mitigates Co′s negative effects, suggesting that Fe and Zn offer protective benefits. The specific effects of these metals on *Nitrosopumilaceae* remain unclear due to the lack of culture‐based studies (Herber et al. 2020). *Woesearchaeales*, associated with carbon and hydrogen metabolism in anoxic conditions, increase with Co and mixed treatments, likely due to Co as a cofactor (Huang et al. 2021). *Marine Group III* may also utilise Co, giving them an advantage in metal‐enriched environments (Jose et al. 2017).

### 4.5 Effect of Environmental Parameters on Bacterial and Archaeal Communities

Our statistical analyses using one‐way ANOVA and post hoc Dunnett's test confirm that each micronutrient, whether applied individually or in combination, may exert distinct influences on nutrient levels and microbial populations in both oxygenated and deoxygenated conditions. The significant fluctuations in nutrient concentrations suggest that these trace metals may modulate nutrient cycling processes, potentially through mechanisms such as chelation, adsorption or alterations in microbial activity that impact nutrient availability (Boyd and Ellwood 2010; Morel and Price 2003). The changes observed in TBC indicate that these micronutrients can either promote or inhibit bacterial growth, depending on their concentrations and the specific bacterial communities present.

Analysis of bacterial community composition using CCA revealed a profound influence of micronutrients on bacterial diversity and composition. The distinct clustering of bacterial groups indicates varied species responses to metal exposure, with some groups possibly developing resistance or adaptive mechanisms (Silver and Phung 1996; Emerson, Fleming, and McBeth 2010), while others are more susceptible to metal stress due to sensitivity or lack of adaptation. Similarly, archaeal CCA analysis highlighted nitrate, nitrite and phosphate as critical nutrients influencing the structure of archaeal communities. The presence of silicate and ammonia underscores the diverse ecological niches occupied by different archaeal taxa. These taxa play a crucial role in influencing nutrient dynamics through the decomposition of siliceous organisms, which leads to the release of silicates and ammonia back into the water column, highlighting their importance in deep‐sea ecosystems. Specifically, samples treated with Fe, Zn and Co exhibited enhanced growth of specific archaeal taxa, with clear clustering patterns indicating significant alterations in archaeal community compositions over time. The correlation analysis between environmental variables and microbial diversity underlines how metal amendments influence nutrient dynamics, thereby shaping the ecological dynamics of microbial communities in marine environments, whether oxygenated or deoxygenated.

## 5 Conclusion

Our study underscores the significant influence of micronutrients such as Zn, Fe and Co on the diversity and composition of bacterial and archaeal communities in both oxygenated and deoxygenated marine habitats (Yin et al. 2019). While essential as metalloenzymes (Velasco et al. 2018), imbalances in their concentrations can disrupt microbial community structures (Yin et al. 2019). Variations in nitrate and silicate levels in treated samples highlight distinct ecosystem changes between aerobic and anaerobic environments. We found that microbial growth within the AS‐OMZ is notably affected by the availability of Fe, Co and combined treatment, with Fe playing a critical role in oxygenated waters. Additionally, our study emphasises the significant role of oxygen availability in shaping microbial ecology in marine environments. Furthermore, our research highlights the urgent need for culture‐based studies of diverse bacteria and archaea. Overall, our findings not only highlight the varied impacts of micronutrients on nutrient dynamics and microbial communities under different oxygen conditions but also stress the importance of detailed physiological characterisation of environmental microbes.

## Author Contributions

**Mandar Bandekar:** conceptualization, methodology, investigation, data curation, visualization, writing – original draft, writing – review and editing, supervision. **Rakhee Khandeparker:** funding acquisition, resources, writing – review and editing, validation, project administration. **Kuldeep D. More:** data curation, visualization, writing – original draft, writing – review and editing, software. **Seyieleno C. Seleyi:** formal analysis, data curation, writing – original draft, writing – review and editing. **Mukund Gauthankar:** formal analysis. **Ujwala Amberkar:** formal analysis. **Jukka Kekäläinen:** writing – review and editing. **Jarkko Akkanen:** writing – review and editing.

## Conflicts of Interest

The authors declare no conflict of interest.

## Supporting information


**Data S1.** Supporting Information.

## Data Availability

The data that support the findings of this study are available on request from the corresponding author. .
