# Supplementary material for: Microbial Responses to Micronutrient Amendments in Oxygenated and Deoxygenated Waters of the Arabian Sea
Source: Environ Microbiol Rep. 2025 May 2;17(3):e70072. doi: 10.1111/1758-2229.70072 (PMC12046384; doi:10.1111/1758-2229.70072)
Supplement: Supplementary file 1 — Data S1. Supporting Information. [file EMI4-17-e70072-s001.docx]

**Supporting Information**

**Microbial responses to micronutrient amendments in oxygenated and deoxygenated waters of the Arabian Sea**

Mandar Bandekar^1,2,^*****, Rakhee Khandeparker^2^***,** Kuldeep D. More^3^*****, Seyieleno C Seleyi^4^, Mukund Gauthankar^2,5^, Ujwala Amberkar^2,6^, Jukka Kekäläinen^1^, Jarkko Akkanen^1^

^1^Department of Environmental and Biological Sciences, University of Eastern Finland, Joensuu and Kuopio, Finland

^2^Biological Oceanography Division, CSIR-National Institute of Oceanography, Dona Paula, Goa 403004, India.

^3^Department of Biological Sciences, State University of New York at Buffalo, Amherst. NY 14068

^4^Marine Biotechnology Division, National Institute of Ocean Technology, Ministry of Earth Sciences, Chennai, India

^5^Départment des Sciences Fondamentales, Université du Québec à Chicoutimi, 555, boulevard de I´Université, Chicoutimi (Québec) G7H 2B1, Canada

^6^Enviornmental Impact Assessment Division, National Centre for Polar and Ocean Research, Vasco-da-Gama, Goa, India

**Table S1**. Measures of physical and oxygen parameters from oxygenated (73 m) and deoxygenated (200 m) depths at the Arabian Sea Time Series (ASTS). DO= Dissolved oxygen

| **Depth (m)** | **Temperature (°C)** | **Salinity(PSU)** | **DO (μmol L^−1^)** | | **pH** |
| --- | --- | --- | --- | --- | --- |
| 73 | 27.04 | 36.35 | 203.76 | 8.05 | |
| 200 | 15.28 | 35.72 | 3.47 | 7.76 | |

**Table S2**. Relative abundance percentages and depth wise distribution of top 10 phyla in the initial sample (0) and after five days (day 5) in the microcosms. See Fig. 1 for abbreviations.

|  | **Treatments** | | | | | |
| --- | --- | --- | --- | --- | --- | --- |
| **Bacterial taxa** | **C73-0** | **C73-5** | **Zn73-5** | **Fe73-5** | **Co73-5** | **Mix73-5** |
| Proteobacteria | 43.07 | 42.98 | 50.98 | 52.00 | 50.20 | 43.63 |
| Actinobacteriota | 22.18 | 20.97 | 8.98 | 16.53 | 13.53 | 16.95 |
| Thermoplasmatota | 14.08 | 11.95 | 5.94 | 7.27 | 5.97 | 19.96 |
| Cyanobacteria | 10.01 | 8.00 | 1.96 | 1.37 | 2.75 | 3.91 |
| SAR324 clade | 3.07 | 3.48 | 3.92 | 8.98 | 2.92 | 4.98 |
| Marinimicrobia | 2.14 | 2.94 | 1.95 | 3.96 | 0.92 | 3.99 |
| Crenarchaeota | 2.07 | 2.98 | 1.95 | 2.99 | 5.98 | 1.93 |
| Verrucomicrobiota | 1.02 | 2.96 | 0.96 | 1.98 | 4.98 | 0.73 |
| Firmicutes | 1.12 | 1.98 | 0.92 | 1.99 | 0.90 | 0.99 |
| Bdellovibrionota | 1.24 | 1.76 | 22.50 | 2.94 | 11.87 | 2.94 |
|  |  |  |  |  |  |  |
|  | **C200-0** | **C200-5** | **Zn200-5** | **Fe200-5** | **Co200-5** | **Mix200-5** |
| Proteobacteria | 70.2 | 69.46 | 53.98 | 59.94 | 43.98 | 48.98 |
| Actinobacteriota | 10.48 | 9.98 | 16.95 | 18.95 | 9.97 | 10.96 |
| Bdellovibrionota | 1.98 | 1.99 | 0.79 | 2.28 | 4.96 | 0.94 |
| SAR324 clade | 5.06 | 4.93 | 1.86 | 3.26 | 2.99 | 2.99 |
| Crenarchaeota | 5.04 | 4.99 | 3.98 | 2.22 | 3.99 | 2.94 |
| Marinimicrobia | 3.06 | 3.98 | 4.94 | 8.34 | 22.99 | 15.33 |
| Firmicutes | 2.08 | 2.95 | 2.98 | 1.48 | 1.73 | 2.97 |
| Patescibacteria | 1.21 | 0.97 | 13.92 | 2.56 | 0.42 | 12.96 |
| Nanoarchaeota | 0.89 | 0.84 | 0.93 | 0.98 | 8.97 | 1.95 |


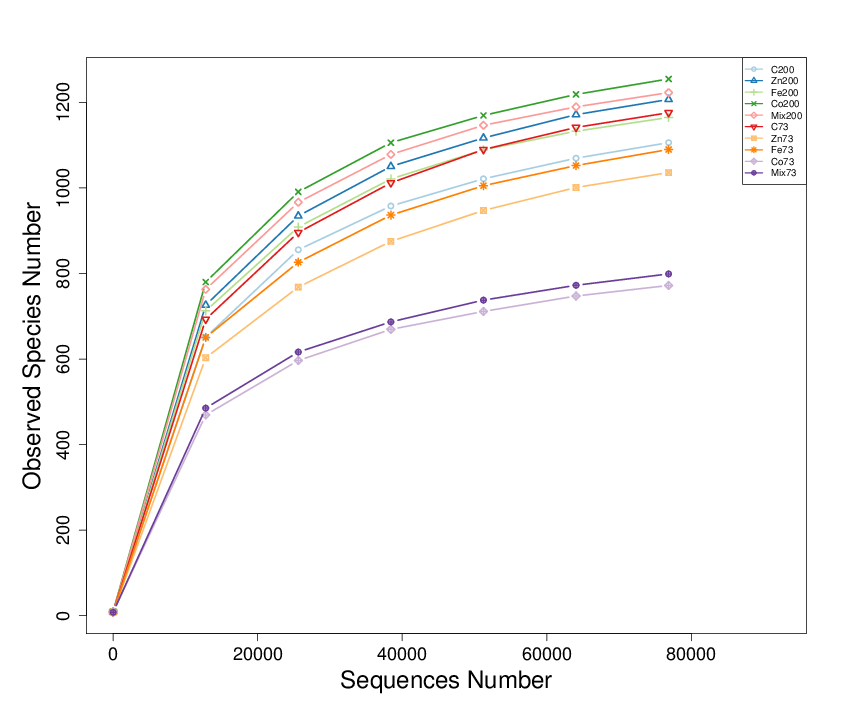


**Figure S1.** Rarefaction analysis of total microbiota from different micronutrient treatments (Zn, Fe, Co, and mix) from oxygenated (73 m) and deoxygenated (200 m) depths of the Arabian Sea Time Series (ASTS) location.
